# A Systematic Review of Instruments to Assess Guilt in Children and Adolescents

**DOI:** 10.3389/fpsyt.2020.573488

**Published:** 2020-12-09

**Authors:** Vittoria Zaccari, Marianna Aceto, Francesco Mancini

**Affiliations:** ^1^Associazione Scuola di Psicoterapia Cognitiva (APC – SPC), Rome, Italy; ^2^Department of Human Sciences, Marconi University, Rome, Italy

**Keywords:** guilt, development, measure, instrument, children, adolescents, systematic review

## Abstract

**Background:** Guilt feelings have received considerable attention in past psychological theory and research. Several studies have been conducted that represent a range of views and propose various implications of guilt in children and adolescents. Variations in theoretical definitions of guilt, emphasizing a lack of measurement convergence, make it difficult to derive a comprehensive definition of the construct in childhood and adolescence. Research shows substantial variability in instruments used to measure guilt in children and adolescents.

**Purpose:** The aim is to discuss existing contributions, illustrating the empirical validity of the available instruments used to measure guilt and identifying the nature of their theoretical backgrounds among children and adolescents.

**Methods:** A systematic search was conducted using the following databases: PsycINFO, PsycARTICLES, MEDLINE, Scopus, Web of Science, and PubMed (all years up to February 19, 2020). Search terms were compiled into three concepts for all databases: “measure,” “guilt,” and “childhood/adolescence.” In addition, a search was conducted to detect the gray literature.

**Results:** After removing the duplicates, a total of 1,408 records were screened, resulting in the identification of 166 full-text articles to be further scrutinized. Upon closer examination, there was consensus that 148 of those studies met the study inclusion criteria or were not retrieved. Twenty-five studies were included in the quality assessment. The data were organized on three main categories: (1) interpersonal or prosocial guilt; (2) intrapunitive guilt or that referring to an excessive sense of responsibility; (3) not specifying a theoretical construct. A great heterogeneity in psychometric evaluations and substantial variability in guilt construct emerged. The construct most represented and supported by valid instruments was interpersonal or prosocial guilt. Analysis of the gray literature showed that some instruments were not immediately available to the clinical and scientific communities.

**Conclusions:** The studies analyzed and selected for qualitative review employed various instruments to measure guilt. Results confirmed what is widely documented in the literature about substantial variability in instruments used to measure guilt. We argue the need to develop measures that assess currently overlooked dimensions of guilt and to provide further additional information about the psychometric proprieties of the available developed instruments.

## Introduction

### Guilt Feelings: Theoretical Approaches

Guilt feelings have received considerable attention in past psychological theory and research. This is not surprising, since they have been considered to be a key element within the human moral and social experience. Given its role in mediating the relationship between moral internalized intentions and moral behavior, guilt has been defined as a moral or self-conscious emotion (1–4). An often cited definition of guilt considers it as a painful emotion that arises when an individual causes or believes he/she has caused harm to another; in other instances, there may be a violation of moral or social norms or personal internalized values (3, 5, 6). Experience of guilt would inherently involve a sense of responsibility for a transgression's outcome (7) and is considered to promote an other-oriented reparative attitude that is motivating individuals to accept responsibility and take reparative action in the wake of the occasional failure or transgression (4).

However, across the literature one finds a range of approaches to the whole notion of guilt, highlighting some contradictory premises about its nature and genesis. Historically, psychoanalytic theorizing played a pivotal role in the psychological study of guilt, describing it as a self-punitive process that takes place within the individual by sanctioning or censuring violations of moral standards (2). This process disposes the individual to actions aimed at reducing his/her personal discomfort—regardless of the potential to repair the damage producing the internal suffering (2). The psychodynamic approach (8–17) developed within an intrapsychic perspective has focused on the negative characteristics of guilt, emphasizing its involvement in the development and maintenance of psychopathology. This approach describes guilt by characterizing it as not necessarily related to others. Guilt feeling is an internal conflict associated with a violation of internalized norms, and not necessarily related to another's suffering (3).

Other psychological approaches (2–4, 18, 19), developed within an interpersonal perspective, have assigned an adaptive role to guilt, showing its motivational drive toward prosocial, altruistic behaviors and the development of an empathic preoccupation for the well-being of others. This approach argues that feelings of guilt derive from the perception of having harmed or not given help to another person, and therefore the guilt is felt not toward an internalized authority but toward another person. This perspective attributes an influential role to empathy (20).

In recent years, Mancini and Gangemi (21) have suggested a dualistic model of guilt in adulthood [see also (22)] that considers its multidimensional nature. In this dualistic model, two distinct senses of guilt, such as altruistic guilt and deontological guilt, can occur simultaneously, and they derive, respectively, from damage to others or from a violation of a moral norm (with no necessary involvement of a victim). As such, this model begins to reconcile the various perspectives on this emotion (3, 23). In this evidence-based construct, intrapsychic and interpersonal models are not mutually exclusionary; rather, they are oriented toward two distinct emotions, such as deontological guilt and altruistic guilt, which differs in relation to the appraisal of an event in the context of individual goals, desires, and beliefs. The two senses of guilt are not activated by different types of event but differ only by virtue of the interpretation of an event in the context of individual goals, beliefs, or desires. In altruistic guilt there is always a victim suffering harm and the belief of not having been altruistic. This emotional state is strictly connected to empathy and the goal of altruism, soliciting altruistic attitudes in the attempt to expiate errors or deficits. In deontological guilt there could be no victim at all (e.g., incest between consenting siblings), but there is the assumption of having violated the “Do not play God” principle (23).

These main conceptualizations contain fundamental and substantial differences. Guilt is conceptualized as other-focused and self-focused emotion. The first is focused on the other and related to the empathic care for suffering inflicted on the other. The second is focused toward itself with respect to the transgression of an internal norm.

These conceptualizations emphasize an involvement of morality in defining guilt, and they broadly diverge in considering guilt either inherently adaptive or maladaptive (6).

### Clarifying the Nature of Guilt: A Central Clinical Issue

Thus far, most research on guilt and psychopathology has been conducted with adult populations. It was found that various clinical manifestations are linked to different types of guilt.

This is evident from the description in the Diagnostic and Statistical Manual of Mental Disorders [DSM-5; (24)] that emphasizes the role of excessive or abnormal guilty feelings in some mental disorders, such as major depressive episodes, post-traumatic stress disorder, and depression (25, 26), as well as in obsessive-compulsive disorder [OCD; (27)]; and a lack of guilt or remorse in conduct disorder and antisocial disorder (28). Some studies (29–31) have supported a connection between proneness to guilt and eating disorders.

The literature documents that some types of guilt are characteristic of some clinical phenotypes. For example, a review by Gangemi and Mancini (32) suggested that guilt in OCD is predominantly deontological. In particular, research on OCD has found that abnormal feelings of guilt and responsibility are the typical cognitive features of this clinical profile. A strong sensitivity to guilt is considered a basic element in the pathology of OCD, particularly in cognitive-behavioral models (27, 33–36). Also, a positive and significant association was found between depressive symptoms and altruistic guilt or high levels of empathy (37, 38). These results suggest that some psychopathological disorders are characterized by specific guilt feelings.

### Guilt: Maladaptive Behaviors and Clinical Manifestations in Children and Adolescents

Several studies on children and adolescents have showed that guilt feelings play a role in adaptive and maladaptive behaviors, highlighting significant correlations with different symptomatologic manifestations. Malti and Krettenauer (39) reported a positive link between guilt feelings and prosocial behavior. There is also some support for a positive association between guilt and reparative behavior, both in early childhood (40) and in mid-childhood and adolescence (41, 42). Also, in a recent study, Vaish et al. (43) showed that from early in childhood, at least 3 years of age, guilt promotes prosocial reparative behavior.

Despite empirical support for the potential link between guilt and prosocial adaptive behavior [and thus a guilt-engendered positive outcome; see (44)], several studies have pointed toward a relationship between guilt and a range of psychological disorders (45, 46). In fact, some authors [e.g., (39)] have suggested that guilt feelings have implications for both psychopathology and healthy outcomes in children and adolescents. Two reviews (39, 47) highlighted a negative relation between guilt and aggression in childhood and adolescence, regardless of age reporting; in contrast, a positive link was found between guilt feelings and prosocial behavior. Other studies (39, 48) have suggested that very low levels of moral guilt and disregard for others are positively associated with aggressive and antisocial conduct in early childhood (49), middle childhood (50), and adolescence (51). A study by Colasante et al. (52) tested guilty feelings as potential moderators of the daily anger deviations aggression link, highlighting how aggravating the link between daily anger deviations and aggression was weaker for children with relatively high levels of guilt.

Importantly, researchers have argued that there is a close link between the absence of moral guilt and aggression, violence, and antisocial conduct, both in the normative and clinical range (underscoring the importance of guilt in predicting aggressive conduct in normative samples throughout development). This finding is reflected in the inclusion of guilt in the diagnostic classification of externalizing disorders, such as conduct disorder (CD), in the DSM-5 (24). Similarly, excessively high levels of neurotic guilt, representing an inappropriate context for guilt, may be directly or indirectly related to internalizing symptoms across childhood and adolescence, such as feelings of hopelessness, low self-efficacy, and self-depreciation (53).

Furthermore, Reeves et al. (54) documented in children preliminary results that provide support for a link between inflated responsibility (an excessive obligatory sense in which the individual evaluates his/her own thoughts in terms of the harm they could cause to themselves or others) and increased checking behaviors; this view is in line with the inflated responsibility model of OCD (33), as may be applied to children.

However, although some recent clinical-developmental perspectives (4, 5, 7) have pointed out potential maladaptive outcomes of excessive or inappropriate feelings of guilt, there are few studies exploring the specific implications of abnormal guilt feelings in evolutive psychopathology. There appears to be greater confusion for children and adolescents about the different dimensions of guilt involved in individual disorders.

These results highlight a lack of conceptual convergence on a common underlying construct of guilt in the present literature. Understanding the role of guilt seems to be complicated by the heterogeneity of the construct and the way it is operationalized (6). Probably this is due to the apparent lack of guilt assessment tools that clearly define the nature of the construct they are intended to measure. This represents an important limitation for childhood and adolescence psychopathology, as it is not noted in the literature what is suggested by high or low levels of guilt, in their specific dimensions, with respect to the different clinical phenotypes.

These results reveal a variability in the definition of guilt, and the divergent views on its relation to problematic outcomes (6) brings into question its direct link to psychopathology [see also (18–20, 55–58)].

### Guilt Assessment

Given the implication of guilt in psychopathological disease, various instruments have been devised to detect the presence and extent of this condition, especially in adult populations.

Instruments display a broad heterogeneity in the conceptualization and validation of measures of guilt in adults, especially for the variability in the definition and measure of guilt as a state or trait-like component, an involvement in blaming toward the self or the behavior, and the relation with other overlapping constructs. For example, the Guilt Inventory [GI; (59)] is an evaluation scale that separately measures trait guilt, state guilt, and moral standards. It is based on the concept that guilt is an emotion that does not coincide with moral standards and therefore requires separate measurement instruments. The Interpersonal Guilt Questionnaire-67 [IGQ-67; (60)] evaluates four different types of guilt and measures them separately: survivor guilt, separation guilt, omnipotent responsibility guilt, and self-hatred guilt. The Guilt and Shame Proneness (GASP) scale measures individual differences in the proneness to experience guilt and shame across a range of personal transgressions (61). Global adjective checklists such as the Personnel Feelings Questionnaire-2 [PFQ-2; (62)] assess trait guilt by asking respondents to rate the frequency with which they experience guilt-related adjectives. The Test of Self-Conscious Affect [TOSCA−3; (63)] yields indices of shame-proneness, guilt-proneness, externalization, detachment/unconcern, Alpha pride, and Beta pride.

Several studies have been conducted to identify and measure guilt in childhood and adolescence. However, a narrative review by Tilghman-Osborne (7) showed that, in children and adolescents, measures of guilt have mostly been focused on the adaptive aspect of the construct, attitudes toward reparation, and feelings of responsibility. Such positive aspects of guilt are less evident in theories and measures developed for older populations. Specifically, it was detected that within existing measures, behavioral coding and guilt induction strategies are most widely used in children younger than 5 years of age, whereas questionnaires or interviews are more commonly used for children older than 5 years. Within the most widely used instruments, there are the scenario-based self-report measures of characteristics of shame and guilt-proneness, e.g., the Test of Self-Conscious Affect for Children (for 8- to 12-year-olds) and the Test of Self-Conscious Affect–Adolescent [TOSCA-C/A; (64, 65)] (for 12- to 20-year-olds). Other instruments include semi-projective measures such as Hoffmann's Stories (66) or behavioral measures such as the Doll Paradigm (67).

In the overview of instruments to identify and measure guilt, self-report measures predominate in older children and adults and self-report measures are more used than interviews and questionnaires (6). These instruments highlight how often the construct of guilt is not always defined and homogeneous. Heterogeneity is found above all in developmental psychology research in children and adolescents, which has been focused in particular on the adaptive aspect of the guilt construct and shame (4, 68). Historically, the distinction between shame and guilt, two self-conscious emotions, has not been emphasized, and numerous researchers have used these terms interchangeably (69). Guilt and shame are hardly to measure as separate constructs, probably since they share, in certain conditions, similar features, and because they frequently co-occur. However, research has noted theoretical differences between the two emotions, and guilt and shame are now consistently discussed as separate constructs (2, 4, 5). Specifically, guilt reflects a more negative appraisal of one's particular behavior, decentering from the self and focusing attribution on the wrongness of a specific behavior (4, 70).

Therefore, clinical research and practice show substantial variability in instruments used to measure guilt, particularly in children and adolescents. This heterogeneity is likely due to the variant theoretical models on which they are constructed and/or the specific features of guilt emphasized by a given set of researchers (6, 71).

### Rationale

The different theoretical approaches and the different types of guilt detected by instruments highlight that guilt is a multidimensional construct (3, 21). Currently, these substantial variations in theoretical definitions of guilt, emphasizing a lack of measurement convergence, make it difficult to derive a comprehensive definition of the construct in childhood and adolescence (6). This underlines the importance of differentiating the type of guilt in children and adolescents, and given the theoretical heterogeneity of the construct of guilt, it is important for researchers and clinicians to know which evaluation instruments are described in the literature and on which aspects of the construct they are based. However, the theoretical distinctions of guilt have a clinical importance, as demonstrated in particular by studies on adults. Even in childhood and adolescence it is necessary to have tools that are able to distinguish different types of guilt. At present, the assessment of guilt in children and adolescents appears scattered and unclear, as suggested by the narrative review of Tilghman-Osborne et al. (6). A systematic analysis of how guilt is detected with specific validated psychometric instruments in children and adolescents is lacking. These reflections indicate the need to fill this gap in the literature. Our systematic review allows us to offer a complete overview of the state of the art and to outline future developments of this line of research. It is essential to know which instruments were validated or developed, and to verify if they are based on a reference theory and if information is available to evaluate the instrument.

### Research Question

The aim of this review is two-fold: (1) to review the existing contributions illustrating the empirical validity of the available instruments, for both researchers and clinicians, used to measure guilt among children and adolescents; (2) to identify the nature of these instruments' theoretical backgrounds and especially to determine whether some types of guilt are overrepresented in the assessment literature and, conversely, if other guilt subtypes are overlooked and need further attention by psychometric researchers. Of note, this study does not aim to draw conclusions about the psychometric proprieties of available instruments. Rather, we were interested in providing a systematic view on the existing lines of research in the field, in order to draw epistemologically grounded conclusions and recommendations for future research. It is important, even during an individual's childhood and adolescence, to identify and delineate the nature and degree of regulatory processes that undergird both adaptive guilt and that which is atypical and maladaptive. This can lead to a more comprehensive understanding of the condition that can arise during the most formative period of a person's life. Such an understanding can also contribute to more appropriate interventions and, further, help determine the degree to which guilt may be present in and help drive a range of psychopathologies that can afflict children and adolescents.

## Method

**Figure 1:**
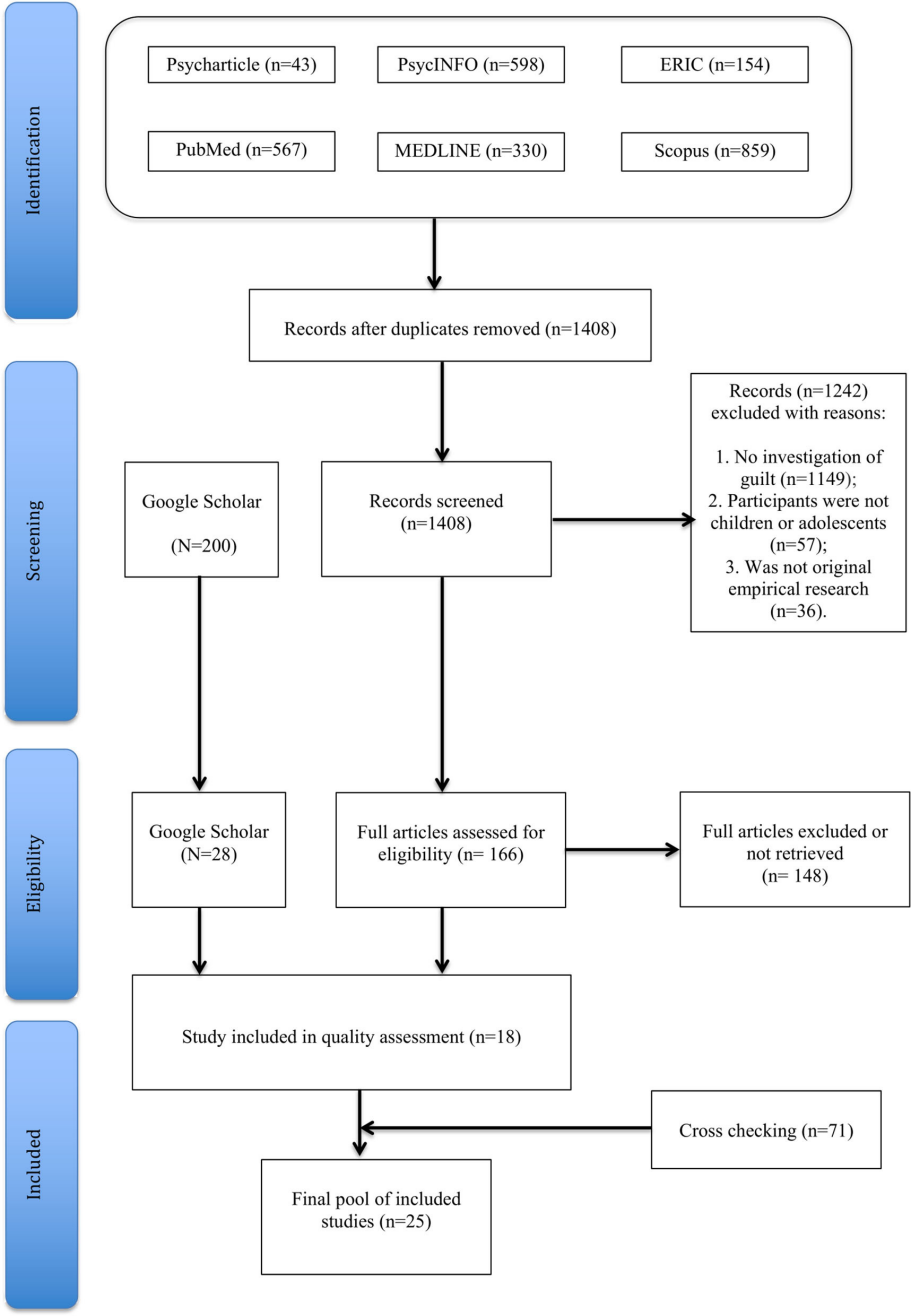
Flow diagram describing the processes of identification, screening and inclusion of the studies.

A systematic search was conducted according to the PRISMA guidelines (72). The flow diagram depicted in Figure 1 illustrates the entire process of study identification and selection (based on the inclusion criteria used; see below).

### Eligibility Criteria

The inclusion of studies in the systematic review was decided according to the following criteria: (1) The first aim of the study was to illustrate and/or validate an instrument assessing guilt; (2) the instrument was a self-report questionnaire or an interview; (3) the instrument was used to measure guilt in a population of children and/or adolescents; (4) the instrument measured generalized guilt; and (5) the design of studies was cross-sectional or longitudinal. Exclusion criteria were: (1) not providing original contributions (e.g., review, comment, or letter to the editor); (2) providing exclusively qualitative data; (3) studies having been conducted on a population 18 years of age or older; (4) studies examining exclusively contextual guilt. Published status and language of the contribution were not exclusion criteria, nor were gender composition, ethnicity, nationality, and clinical status of the sample.

### Search Strategy

The literature search was conducted using the following databases: PsycINFO, PsycARTICLES, MEDLINE, Scopus, Web of Science, and PubMed (all years up to and including February 19, 2020). Search terms were aggregated into three concepts for all databases: “measure,” “guilt,” and “childhood/adolescence” (see Appendix A in Supplementary Material).

Regarding the “guilt” concept, to elaborate the search syntax, several methods have been used, with the aim of being as exhaustive as possible. First, we searched for relevant systematic reviews and meta-analyses published on the topic of guilt and examined the search terms used by the authors in some primary studies. Then, a search in the thesaurus of the mesh terms database was performed. Finally, authoritative narrative reviews shedding light on the heterogenous nature of the concept of guilt [e.g., (6)] were examined, and guilt-related terms were extracted and added to the list of our search terms. Too broadly related terms, including a wider array of emotional states (e.g., “negative emotionality,” “self-conscious emotions”) were excluded. Similarly, terms related to psychopathological conditions that are thought to be related to guilt (e.g., callous-unemotional, alexithymia) but are not directly expressing the concept of guilt were excluded. In relation to the “measure” concept, as we were interested in collecting contributions that offer empirically validated instruments to both the scientific and the clinical community, we included terms related to common procedures to measure psychological variables in the field, such as questionnaires and interviews. Despite the insightful nature and utility of the experimental procedures used to assess guilt, we decided to exclude this type of assessment because they are too remotely related to a potential clinical application. Finally, the concept of “childhood and adolescence” was added to ensure that the search would have been restricted to our field of interest, namely, developmental psychology.

In addition, we performed a search of the gray literature following three strategies. First, we carried out a search on Google Scholar on 01/09/2020 using search terms related to our three concepts (see Appendix A in Supplementary Material for the detailed search syntax). As suggested by Haddaway et al. (73), we screened the first 200 results for pertinent studies. Moreover, all the reference lists of included studies as well as authoritative narrative reviews on the topic [e.g., (6)] were screened for additional studies. When the gray literature was unavailable, we contacted the authors by sending an e-mail, asking for the full text of the contribution. Finally, we wrote 18 e-mails to the main authors of the field, asking for unpublished data on the topic.

### Selection of Studies

We screened every title and abstract to determine the eligibility of the study for inclusion. Two reviewers (VZ and MA) independently conducted the electronic searches using the aforementioned databases. Together, independent review of these electronic databases identified a total of 2,551 articles with the initial search terms, which were then examined by each reviewer for eligibility. After removing the duplicates, a total of 1,408 records were screened, resulting in the identification of 166 full-text articles to be further scrutinized. Of these, 16 were retained for inclusion in the present study. In addition, another 28 full-text articles from Google Scholar and 71 from the cross-checking procedure were examined, leading to the inclusion of nine additional papers. A total of nine unpublished contributions were not retrieved despite having sent an e-mail to the authors. The list of this gray literature can be found in Appendix A (Supplementary Material).

Then, a coding protocol was developed that allowed for the extraction of the following information: (1) metadata (i.e., authors, year of publication, publication status of the contribution); (2) information related to the sample used in the studies (i.e., mean age or age range, sample size, gender composition, and clinical status); (3) methodological information of the studies (the nature of the instrument used and the research design); and (4) contents information provided by the studies (theoretical guilt-related framework, type of psychometric information provided).

## Results

A total of 25 studies were selected for qualitative review. As the main goal of our study was to illuminate the theoretical background underlying the development of instruments measuring guilt in a population of children or adolescents, we classified each study according to three main categories based on the type of conceptualization of guilt used by the author(s): interpersonal or prosocial guilt; intrapunitive guilt (i.e., an excessive sense of responsibility); and studies not specifying any underlying theoretical construct or, alternatively, using a conceptualization not classifiable within the two former categories. The decision to classify the studies within these categories arose from a general examination of the studies and from reflection among all authors about the most frequent redundancies in the theoretical background cited by the studies.

### Studies Adopting the Construct of Interpersonal or Prosocial Guilt

As illustrated in Table 1, a total of seven studies adopted the construct of interpersonal or prosocial guilt as the theoretical framework underlying the instrument. All articles had been published within the last two decades, and most of the studies had been conducted on a non-clinical population. Sample sizes were adequate, with the number of participants ranging from 50 (74) to 699 (70). Also, the gender composition of the sample was almost balanced. Regarding age, it should be noted that most of studies had been conducted on a sample of adolescents, or in any case not on participants younger than 7 years of age. Regarding the instrument's characteristics, all studies used a self-report questionnaire. In addition, the TOSCA (or adapted versions) was used in the majority of these studies. Finally, all studies provided some kind of psychometric information, but only studies using the TOSCA ran factorial analyses to test the underlying structure of the instrument.

**Table 1:** Studies using measures of interpersonal or prosocial guilt among sample of children or adolescents.

**References**	**Country**	**Sample**			**Instrument**	**Type of instrument**	**Design of research**	**Psychometric analyses performed**
		***N* and nature**	**Age (years)**	**% males**				
Kronmüller et al. (74)	Germany	505 NC 50 C	8–18	46.2	German TOSCA-C/A	Self-report	Cross-sectional	•Internal consistency •Reliability •Intercorrelation between scale
Laskoski et al. (75)	Brazil	580 NC	16 (1.19)	44.4	ESCA	Self-report	Cross-sectional	•Internal consistency •Convergent/discriminant validity
Benesch et al. (76)	Germany	131 C	8.9 (1.9)	ns	ICU	Self-report	Cross-sectional	•Internal consistency •Convergent/discriminant validity
Watson et al. (77)	Australia	562 NC	13.4 (0.92)	43.06	TOSCA-A	Self-report	Cross-sectional	•Factorial structure and its invariance across gender •Internal consistency •Convergent/discriminant validity
Watson et al. (70)	Australia	699 NC 562 NC	13.41 (0.92)	47.5 43.1	TOSCA-A	Self-report	Cross-sectional	•Factorial structure •Internal consistency •Convergent/Discriminant validity
Shahnawaz and Malik (68)	Pakistan	459 NC	16.4 (1.3)	22.2	TOSCA-A	Self-report	Cross-sectional	•Factorial structure •Internal consistency •Predictive validity
Broekhof et al. (78)	Netherlands	225 NC 108 C	11.62 (1.37)	42.2	BSGQ	Self-report	Cross-sectional	•Internal consistency •Convergent/Discriminant validity

*NC, non-clinical sample; C, clinical sample; TOSCA-C/A, Test of Self-Conscious Affect for Adolescents/Children; ESCA, Guilt Scale for Adolescents; ICU, Inventory of Callous-Unemotional Traits; BSGQ, Brief Shame and Guilt Questionnaire*.

### Studies Adopting the Construct of Intrapunitive Guilt or Guilt Conceptualized as an Excessive Sense of Responsibility

Ten studies were framed within the conceptualization of guilt as an intrapunitive process or as an excessive sense of responsibility. Their main characteristics are displayed in Table 2. The date range in this category is wide, extending from 1977 to 2019. The majority of these studies were conducted in the US or in Europe. Except for the study of Bacow et al. (83), all studies were conducted on wide sample sizes, ranging from 121 to 6,709 participants. Of note, only one of the investigations included a clinical group in the sample (83), and none of the examined studies was conducted with participants below 7 years of age. Despite the general heterogeneity of the results, it should be noted that each of them used a self-report questionnaire. Four studies examined the validity of the self-blame subscale of the CERQ, and two focused on the same SRP subscale. Regarding the type of psychometric evaluations provided by the studies, a great heterogeneity emerged, with some authors only providing data related to the internal reliability of the instrument (79) and others performing a wide range of statistical analyses to test the internal consistency, factorial structure, test–retest reliability, and construct validity of the instrument [e.g., (84)].

**Table 2:** Studies measuring excessive sense of responsibility and culpability among adolescents or children population.

**References**	**Country**	**Sample**			**Instrument**	**Type of instrument**	**Design of research**	**Psychometric analyses performed**
		***N* and nature**	**Age (years)**	**% males**				
Henderson (79)	Australia	6,709 NC	13–18	47.53	Subscales of the HDHQ	Self-report	Cross-sectional	•Internal reliability
Cole et al. (80)	USA	121 NC	9–14	49	WIH questionnaire	Self-report	Cross-sectional	•Internal consistency •Convergent validity
Cartwright Hatton (81)	UK	177 NC	13–17	43	SPR subscale	Self-report	Cross-sectional	•Internal consistency •Internal reliability •Predictive validity
Garnefski et al. (82)	UK	717 NC	9–11	39.4	Self-Blame subscale of the CERQ	Self- report	Cross-sectional	•Internal consistency •Convergent/Discriminant validity
Bacow et al. (83)	USA	78 C 20 NC	11.86 (3.11) 12.41 (3.02)	29 7	SPR subscale	Self-report	Cross-sectional	•Internal consistency •Convergent validity •Predictive validity
Tilghman-Osborne et al. (7)	USA	370 NC	10.3 (2.0)	42	IEGS	Self-report	Cross-sectional	•Factorial structure •Internal consistency •Predictive validity
Liu et al. (84)	China	1,403 NC	9–11 (0.87)	52.5	Self-Blame subscale of the CERQ	Self- report	Cross-sectional and longitudinal	•Internal consistency •Reliability •Factorial structure •Test–retest reliability •Predictive validity
García-Vázqeuz et al. (85)	Mexico	661 NC	10.51 (0.64) Girls 10.60 (0.68) Boys	52	DMAE	Self-report	Cross-sectional	•Internal validity •Factorial structure and its invariance across gender •Convergent validity
Orgilés et al. (86)	Spain	582 NC	7–12 (1.2)	51.4	Self-Blame subscale of the CERQ	Self-report	Cross-sectional	•Internal consistency •Test-retest reliability •Predictive validity
Orgilés et al. (87)	Spain	654 NC	9.49 (1.2)	52.1	Self-Blame subscale of the CERQ	Self-report	Cross-sectional and longitudinal	•Internal consistency •Test-retest reliability •Divergent validity •Predictive validity

*C, clinical sample; NC, non-clinical sample; HDHQ, Hostility and Direction of Hostility Questionnaire; WIH, Why It Happened Questionnaire; SPR subscale: Superstitious, Punishment, and Responsibility beliefs subscale of the Metacognitions Questionnaire for Children; CERQ, Cognitive Emotion Regulation Questionnaire; IEGS, Inappropriate and Excessive Guilt Scale; DMAE, Moral Disengagement Scale for Children in Bullying Situations*.

### Studies Not Classified in the Two Former Categories

The main characteristics of the eight studies classified in this category can be found in Table 3. Of note, nearly half of these studies had been published more than 25 years ago. The oldest study in this category was also the only one to use a projective measure (88). Sample sizes were likely to vary, but all were adequate. In addition, the gender composition of the samples was balanced except in the study of Saklofske and Schulz (91), which exclusively recruited males. Each study examined a different instrument. Importantly, the nature of the feelings of guilt was likely to be vague and remained unexplained in almost all studies. Finally, these studies generally provided little psychometric information toward the instrument with, for instance, only two studies testing the factorial structure of the instrument used (89, 95).

**Table 3:** Studies examining guilt among children and adolescent population without referring to interpersonal and intrapunitive guilt.

**References**	**Country**	**Sample**			**Dimension of guilt**	**Instrument**	**Type of instrument**	**Design of research**	**Psychometric analyses performed**
		***N* and nature**	**Age (years)**	**% males**					
Johnson and Kalafat (88)	USA	40 C (hospitalized)	ns	57.5	ns	TAT-type pictures	Projective	Cross-sectional	•Convergent validity
Cattel et al. (89)	UK	800 NC	13.6 (ns) Girls 14.3 (ns) Boys	50	ns	HSPQ	Self-report	Cross-sectional	•Factorial structure
Schuck et al. (90)	USA	85 C	15.7 (ns)	ns	Hostile, sexual and Morality-conscience guilt	FCGI	Self-report	Cross-sectional	•Convergent/discriminant validity
Saklofske and Schulz (91)	USA	77 NC	16.0 (0.94)	100	Guilt state ns	9 guilt adjectives adapted from Haefner	Self-report	Cross-sectional and longitudinal	•Test-retest reliability •Factorial structure
Mathiesen et al. (92)	USA	239 NC	18.75 (2.61)	33	ns	MAAS	Self-report	Cross-sectional	•Internal consistency •Inter-scales correlations
Chung et al. (93)	China	662 NC	14.45 (0.90)	48.2	Self-criticism	4 items of the RSTD	Self-report	Cross-sectional	•Predictive validity
Novin and Rieffe (94)	UK	219 NC	9–14	53	ns	BSGQ-C	Self-report	Cross-sectional	•Internal consistency •Convergent validity
Tani et al. (95)	Italy	242 NC	8–11	54.54	ns	GFS-C	Self-report	Cross-sectional	•Factorial structure

*C, clinical sample; ns, not specified; NC, non-clinical sample; NS, not significant; TAT, Thematic Apperception Test; HSPQ, High School Personality Questionnaire; FCGI, Forced Choice Guilt Inventory; RSTD, Rapid Screening Test for Depression; MAAS, Multidimensional Adolescent Assessment Scale; BSGQ-C, Brief Shame and Guilt Questionnaire for Children; GFS-C, Guilt Feeling Scale for Children*.

## Discussion

### Main Findings

Over the years, numerous investigations have endeavored to identify and measure guilt as it may manifest during the stages of development. However, a systematic analysis of how guilt in children and adolescents has been conceptualized, and the specific instruments applied to determine its presence and extent, has thus far been lacking. The aim of this study was to review the existing contributions, illustrating the empirical validity of the available instruments used to measure guilt among children and adolescents and identifying the nature of their theoretical backgrounds.

Across 25 studies selected for qualitative review, it was found that researchers had applied a broad range of tools. Of the 19 relevant tools detected, all were validated and available in the literature, and two of them had a cultural adaptation. Relatedly, instruments focused on diverse dimensions of the construct of guilt in stages of development, underpinning a heterogeneous theoretical background. For this reason, we classified each study according to three broad categories that captured the dimensions of the construct of guilt, represented in the evaluative instrument(s) for a given study: interpersonal or prosocial guilt (see Table 1); intrapunitive or guilt as excessive sense of responsibility (see Table 2); and other studies that had not specified a theoretical construct or that used a conceptualization not classifiable within the two former categories (see Table 3). Results led us to three observations.

### Measurement Features

First, the tools detected were validated and developed over a long period. The most recent tools, relating to the last two decades, concern those studies that used measures of two main categories of guilt: interpersonal or prosocial guilt and excessive sense of responsibility and culpability (see Tables 1, 2). However, within these two categories, most of the studies had been conducted on a non-clinical population, aged 8–16 years, and had no participants below 7 years of age. All studies used a self-report questionnaire and a cross-sectional design.

### Information on Empirical Validity of Available Instruments

Second, regarding the type of psychometric evaluations provided by the studies, a great heterogeneity emerged. Some authors provided data only related to the internal reliability of the instrument, while others performed a wide range of statistical analyses to test the internal consistency, factorial structure, test–retest reliability, and construct validity of the instrument.

Specifically, concerning interpersonal or prosocial guilt, only studies that used the TOSCA (or adapted versions) ran factorial analyses to test the underlying structure of the instrument. Results evidenced the use of the TOSCA-A (65) or an adapted version in four of the studies that provided information about a wide range of psychometric aspects such as the factorial structure and its invariance across gender and internal consistencies. This may explain why the TOSCA-A is one of the most widely used instruments in studies investigating interpersonal guilt. Relative to the construct of intrapunitive guilt or guilt conceptualized as an excessive sense of responsibility, again, most of the studies showed a great heterogeneity regarding the typology of psychometric evaluations performed. Generally, it appears that most instruments lack replicated information drawn by psychometric evaluations, suggesting the need to further investigate this issue in future studies. Indeed, poor literature related to the topic may lead to overlooking the role played by the intrapunitive guilt construct in children and adolescents psychopathology. To date, the most investigated instruments related to this dimension appear to be the CERQ and the SRP. In particular, four studies examined the validity of the self-blame subscale of the CERQ, referring to thoughts of putting the blame on what an individual has experienced on the self (82, 84, 86, 87), and two focused on the SRP subscale (81, 83), which measures superstitious, punishment, and responsibility beliefs. This instrument has been adapted to different languages and contexts for adolescents and children, and studies have assessed its psychometric properties across countries and different populations.

Regarding instruments with poor theoretical grounding (i.e., our third, “aspecific” category), the studies generally provided little psychometric information, with, for instance, only two studies testing the factorial structure of the instrument used [HSPQ—(89); GFS-C—(95)].

As a whole, psychometric evaluations of some tools were not well-investigated. This indicates the importance of providing information on the psychometric quality of the instruments in children and adolescents. Moreover, the analysis of the gray literature showed that there are several instruments cited in the literature that are not available and do not enjoy empirical validation. This represents a further limitation for the scientific community. This highlighted the need to develop sensitive and reliable measures of guilt among children and adolescents. The availability allows clinicians and researchers to replicate the results obtained from empirical research.

### Instruments' Theoretical Backgrounds

Third, with respect to the nature of instruments' theoretical backgrounds, results confirmed what is widely documented in the adult-related literature about the substantial variability in the guilt construct. This heterogeneity could well be due to the multiple theoretical models on which instruments are built and/or specific features of guilt emphasized by different sets of researchers (6, 7, 71).

In particular, in the third category, there was more evident heterogeneity of the construct. All the studies were based on a poorly defined guilt construct and related to other constructs.

Nevertheless, the results showed which types of guilt are overrepresented in the assessment literature and, conversely, what other guilt subtypes are overlooked. The construct most represented and supported by valid instruments is interpersonal or prosocial guilt detected by TOSCA-A and TOSCA-C. Guilt, in this view, involves a negative evaluation of a specific behavior or action, where the focus is on the wrongness of a particular controllable action. In these instruments, guilt is hypothesized to involve a negative evaluation of the transgressing behavior and to be associated with adaptive and approach responses aimed at repairing (reparation and apology) the consequences of the transgressing behavior (96, 97). An interpersonal perspective assigned an adaptive role to the emotion of guilt by showing its motivational drive toward prosocial behaviors and the development of an empathic preoccupation for the well-being of others (3, 4). Following this, guilt appears to be moderately represented in an intrapunitive dimension according to the intrapsychic perspective.

Although an intrapunitive dimension of guilt was considered in various instruments, there was no specific conceptualization, and guilt appears to be characterized by different definitions. Some instruments detect self-blame (HDHQ subscales) or specifically measure characterological and behavioral self-blame, the former consisting of the attribution of negative events to stable traits or personological dispositions, the latter as an attribution of negative events to specific situational action or inaction (WIH questionnaire). Indeed, intrapunitive behavior has been observed and assessed in various contexts, such as school (80, 98) and in relation to levels of hostility toward the self [(79); HDHQ subscales] and moral disengagement (85). Intrapunitive guilt, though, is not always a factor of the instruments used; often, it only appears as a single subscale (HDHQ, SPR, CERQ subscales).

Indeed, there seems to be no agreement on a definition of intrapunitive guilt, and this dimension could be more challenging to detect than guilt conceptualized on an interpersonal and prosocial level.

Furthermore, results show that guilt conceptualized as an excessive sense of responsibility, due to negative affects and cognitions linked to an erroneous attribution of responsibility, is overlooked. Few instruments detect this dimension.

In the third category, we included all the studies that used an instrument based on a poorly defined guilt construct and related to other constructs. The guilt construct often underlay instruments that evaluate different dimensions and unspecified constructs of hostility guilt, sexual guilt, morality-conscience guilt, and depression. All measures revealed a heterogeneous construct of guilt as their basis.

To identify and measure guilt in children and adolescents, researchers have used various questionnaires and evaluative instruments. Many measures have revealed a heterogeneous construct of guilt as their basis; there also has been a lack of agreement regarding how to identify the condition in children and adolescents. Even within the same categories, the guilt construct was found to be multifaceted. These results suggest that it would be helpful to have, primarily, specific and defined categories of guilt and, as well, instruments capable of distinguishing different types of guilt (and therefore having validity at the construct level). This would help clinicians and researchers verify what high or low levels of guilt suggest in their specific dimensions relative to different clinical phenotypes.

The substantial variations in theoretical definitions of guilt make it difficult to derive a comprehensive definition of the condition in children and adolescents. It is important, even during these years, to understand and identify the amount of developmentally regulatory, adaptive guilt, and atypical, maladaptive guilt. A more complete understanding of the functional and dysfunctional amounts of guilt present at a given time in childhood and adolescence could help clinicians to plan appropriate intervention strategies. One could also determine whether the fault is present specifically in some psychopathologies in children and adolescents. Some research [e.g., (39)] has suggested that guilt feelings have implications for psychopathology and healthy outcomes in children and adolescents. In addition, from the first category, it is underscored that guilt has an adaptive and prosocial nature, while, in the second category, guilt seems to be more related to psychopathological aspects and therefore to a maladaptive aspect of this emotion. This is a limitation, as the lack of clarity of the construct does not allow a clear understanding of which aspect of guilt is actually linked to clinical manifestations in children and adolescents.

Studies that might help to clarify these issues are few; further, the use of instruments is little generalized in other empirical studies. Some studies are quite old and do not clearly specify the construct under investigation. Among the most used instruments currently is the TOSCA (99). Although there is considerable support for Tangney's theory [(18, 96, 97, 100); for reviews, see (101, 102)], alternative models of guilt exist. For example, there are constructs of the emotion that define it in terms of the typology of situations that invoke such responses, often referred to as a public–private distinction (103), wherein guilt is viewed as the result of a private commission of moral transgressions (104, 105).

### Future Directions

However, from the analysis of the construct of guilt among the various instruments collected, the most significant result is a recognition of the heterogeneity of the construct of guilt, which appears confused and various. Guilt can be mainly ascribed to intrapsychic-intrapunitive guilt, interpersonal guilt with a social-adaptive role, and guilt related to other dimensions (3). The heterogeneity of theories regarding the guilt construct appears to be a central and useful issue for future research prospects. Moreover, it constitutes a limit for the development of *ad hoc* validated instruments on specific dimensions of guilt. Mancini's dualistic thesis of guilt (23), focusing on the appraisal process in emotion, could represent an alternative model for conceptualizing guilt. It is supported by research data (22, 106), and it could offer a valid theoretical framework to develop an innovative psychometric instrument for assessing guilt.

These results underline some gaps in the current assessment of guilt in children and adolescents that would benefit from further research. It would be helpful to develop instruments with a psychometric validation and to validate the currently available tools across cultures. It could also be useful to develop behavioral assessment procedures that are more usable and validated for clinical use. Similarly, it would be beneficial to develop instruments able to detect and explore specific typologies of guilt.

### Limitations

This review has some limitations. First, we excluded qualitative measures because we looked for validated instruments and considered only empirical studies. This could be a limitation in that non-empirical studies could be informative. Second, some full texts could not be found, and thus, some instruments may not have been taken into consideration. Third, our literature search did not include unvalidated instruments to assess guilt. Finally, indirect measures such as questionnaires or interviews with parents were not included, nor were physiological measures (sympathetic and parasympathetic activity: SC, skin conductance; RSA, respiratory sinus arrhythmia; HR, heart rate). Studies by Colasante et al. (107, 108) have showed that changes in RSA leading up to and during transgressions were uniquely associated with the intensity of guilt feelings after transgressions in children.

Notwithstanding these limitations, the collective findings in the current study support the conclusion of the heterogeneity of instruments and theoretical underpinnings to detect the guilt construct (6) in the developmental stages. This documents the need to develop instruments to detect other types of guilt and to ensure the availability of studies in the literature.

## Conclusions

Our paper aimed to provide a complete review of instruments investigating guilt in children and adolescents. Importantly, a great number of studies do not use the same theoretical criteria to discriminate the guilt construct. The evaluative instruments described in the literature are variant and are based on different characteristics of the construct and psychometrics. Therefore, using instruments to evaluate guilt could lead to incorrect conclusions, because they are often slanted toward the theoretical model on which they are based. Our results allowing researchers and clinicians interested to the topic to critically appreciate an assessment measure and underline the prospective directions for future research that should aim to make available methodologically sound and theoretically grounded instrument measuring guilt in children and adolescents in order to foster the development of this line of research. This highlights the importance of developing instruments that detect specific features of guilt in terms of both statistical power and goodness of fit, in order to use reliable and valid instruments in the clinic and in research. To improve our knowledge, we need instruments that are reliable in detecting guilt, available for replicating the results obtained from empirical research, and useful for clinicians in order to give clear clinical indications.

## Author Contributions

VZ took overall responsibility for the conceptualization and design of the review and revised it critically for important intellectual content. VZ and MA searched for the articles in the review and assessed them for relevance. VZ, MA, and FM were involved in the interpretation of data, in writing and editing the final article, in approving the final version to be published, in agreeing to be accountable for all aspects of the work, and in ensuring that questions related to the accuracy or integrity of any part of the work are appropriately investigated and resolved. All authors contributed to the article and approved the submitted version.

## Conflict of Interest

The authors declare that the research was conducted in the absence of any commercial or financial relationships that could be construed as a potential conflict of interest.
