# Supplementary material for: A Systematic Review of Instruments to Assess Guilt in Children and Adolescents
Source: Front Psychiatry. 2020 Dec 9;11:573488. doi: 10.3389/fpsyt.2020.573488 (PMC7755888; doi:10.3389/fpsyt.2020.573488)
Supplement: Supplementary file 1 [file Data_Sheet_1.docx]

**APPENDIX A**

TI ( GUILT* OR “SELF-BLAM*” OR “NEGATIVE MORAL EMOTION*” OR “MORAL TRANSGRESS*” OR “SELF-REPROACH*” OR REMORSE OR APOLOG* OR “RESPONSIBILITY” OR “ CULPABILIT*” OR “SELF-PUNISHMENT” ) OR AB ( GUILT* OR “SELF-BLAM*” OR “NEGATIVE MORAL EMOTION*” OR “MORAL TRANSGRESS*” OR “SELF-REPROACH*” OR REMORSE OR APOLOG* OR “RESPONSIBILITY” OR “ CULPABILIT*” OR “SELF-PUNISHMENT”)

AND

TI ( CHILD* OR TEENAG* OR ADOLESCEN* OR YOUNG* ) OR AB ( CHILD* OR TEENAG* OR ADOLESCEN* OR YOUNG*)

AND

TI MEASUR* OR INSTRUMENT OR QUESTIONNAIRE OR INTERVIEW OR TEST OR ASSESSMENT OR VALIDATION

SEARCH TERMS FOR PUBMED DATABASE

(((((MEASUR*[TITLE] OR INSTRUMENT[TITLE] OR QUESTIONNAIRE[TITLE] OR INTERVIEW[TITLE] OR TEST[TITLE] OR ASSESSMENT[TITLE] OR VALIDATION[TITLE])) OR MEASURE[MESH TERMS])) AND ((((CHILD*[TITLE/ABSTRACT] OR TEENAG*[TITLE/ABSTRACT] OR ADOLESCEN*[TITLE/ABSTRACT] OR YOUNG*[TITLE/ABSTRACT])) OR ADOLESCENCE[MESH TERMS]) OR CHILD[MESH TERMS])) AND (((GUILT*[TITLE/ABSTRACT] OR “SELF-BLAM*”[TITLE/ABSTRACT] OR “NEGATIVE MORAL EMOTION*”[TITLE/ABSTRACT] OR “MORAL TRANSGRESS*”[TITLE/ABSTRACT] OR “SELF-REPROACH*”[TITLE/ABSTRACT] OR REMORSE[TITLE/ABSTRACT] OR APOLOG*[TITLE/ABSTRACT] OR “RESPONSIBILITY”[TITLE/ABSTRACT] OR “ CULPABILIT*”[TITLE/ABSTRACT] OR “SELF-PUNISHMENT”[TITLE/ABSTRACT])) OR GUILT[MESH TERMS])

SEARCH TERMS FOR GOOGLE SCHOLAR

(MEASURE OR INSTRUMENT OR TEST OR ASSESSMENT OR VALIDATION) AND (CHILD OR CHILDREN OR ADOLESCENT) AND (GUILT)

Gray literature towards the instruments used to measure guilt in developmental age.

| **Author** | **Study** | **Status of the contribution request** | **Status of the contribution** |
| --- | --- | --- | --- |
| Mulherin,  1998 | Reliability and validity for an adolescent version of the interpersonal guilt questionnaire | Contact not found | Unpublished |
| Haimowitz,  1996 | The assessment of shame and guilt in elementary school children | Contact not found | Unpublished |
| Holland,  1995 | Empirical validation of a model of traumatic guilt | Requested | Not received  Unpublished |
| Stegge and Ferguson,  1994 | Self-conscious emotions: maladaptive and adaptive scales (SCEMAS) | Requested | Not received  Unpublished |
| Bybee, et al.,  1994 | Children’s Guilt Inventory | Contact not found | Unpublished |
| Stegge and Ferguson,  1990 | The Child Version- Child Attribution and Reaction Survey (C-CARS) | Requested | Not received  Unpublished |
| Barrett et al.,  1993 | Doll Paradigm | Contact not found | Unpublished |
| Ferguson,  1996 | My child-shame and my child-guilt | Requested | Not received  Unpublished |
